# ID 362 - Modelo de Curva de Aprendizagem para a Proficiência com Tecnologias Médicas

**DOI:** 10.5327/2237-9622.2025.v34s1.362

**Published:** 2025-11-25

**Authors:** Euler de Vilhena Garcia, Breno Lobo de Almeida

**Keywords:** curvas de aprendizagem, teoria de Vygotsky, modelo cognitivo

## Abstract

**Introdução:**

As curvas de aprendizagem (CA) são modelos matemáticos utilizados para representar o desempenho dos profissionais. Esses modelos se baseiam tradicionalmente na análise da repetição da atividade ou do serviço ao longo do tempo. A diminuição dos tempos relevantes (e.g., tempo de execução, tempo de recuperação...) junto com algumas medidas categóricas (e.g., existência ou não de complicações) é usada como para aprendizado – mas pode refletir apenas que o profissional reage muito bem àquele cenário específico em vez de aprender a fazer, algo como sobreajuste () em treinamentos de máquinas.

Nesse sentido, para demonstrar a eficácia e a eficiência das ações e constatar o processo de aprendizagem do profissional clínico, há a necessidade de integrar um modelo cognitivo que represente a aprendizagem real junto ao modelo operacional que reflita de forma abrangente os resultados obtidos nos treinamentos e procedimentos executados com equipamentos.

**Método:**

Foi realizada uma revisão narrativa de literatura focando o design de equipamentos médicos, o desenvolvimento de tecnologias médicas e a teoria da Zona de Desenvolvimento Proximal de Vygotsky. Foram utilizados termos como "curvas de aprendizagem", "saúde", "tecnologia", "dispositivos médicos" e "ZDP". A partir do levantamento das curvas matemáticas mais utilizadas, seus respect vos contextos de uso e da teoria de ZDP, foi possível definir um modelo matemático capaz de unificar e interpretar os principais tipos de dados coletados em pesquisas de desempenho clínico nesse modelo cognitivo de aprendizado.

**Resultados:**

Apresentamos como é possível abordar curvas de eficiência ou de tempo de execução de tarefas em um modelo cognitivo baseado na teoria de Vygotsky a partir da análise de algumas das principais distribuições utilizadas nas análises quantitativas dos resultados de sobrevida ou de eficiência de execução.

**Conclusão:**

A revisão de literatura ressaltou a abordagem dominante na qual curvas de aprendizagem são frequentemente modeladas de forma operacional e sistemática como análise quantitativa de dados coletados, com critérios principalmente estatísticos de erro. A teoria da Zona de Desenvolvimento Proximal reflete bem a situação de aprendizado clínico em geral – um aprendizado colaborativo, com operacionalização realizada a partir da orientação de companheiros mais capazes na situação –, e seu modelo cognitivo permite análises mais completas dos procedimentos realizados. O modelo matemático aqui apresentado pretende servir de ponte entre os arcabouços teórico das ciências gerenciais, da teoria de aprendizado e dos estudos de desempenho/aprendizagem clínica. Acreditamos que esse aspecto transdisciplinar é fundamental para resolver as atuais limitações de avaliação de desempenho de equipamentos médicos como afetada pela proficiência do usuário operador.

